# Orexin neuron activity in mating mice - a pilot study

**DOI:** 10.35430/nab.2021.e17

**Published:** 2021-06-02

**Authors:** Denis Burdakov, Mahesh M. Karnani

**Affiliations:** 1 Laboratory of Neurobehavioural Dynamics, Institute for Neuroscience, Department of Health Sciences and Technology, ETH Zürich, Zürich, Switzerland^R^; 2 The Francis Crick Institute, London, UK^R^; 3 Institute of Psychiatry, Psychology & Neuroscience, King’s College London, UK^R^; 4 Neuroscience Center Zürich (ZNZ), ETH Zürich and University of Zürich, Zürich, Switzerland^R^; 5 Saints-Pères Paris Institute for the Neurosciences, Université de Paris, France^R^

**Keywords:** Lateral hypothalamus, Mating, Orexin, Hypocretin, Calcium imaging, Miniscope, Freely moving recording, Mouse fellatio

## Abstract

Mating behaviours affect hypothalamic orexin/hypocretin neurons and vice versa. However, activity of orexin neurons has not been recorded during mating before. We report an anecdotal dataset of freely-moving miniature microscope recordings of orexin neuron activity during mating behaviours, as well as an oral sexual encounter previously undocumented in mice. Across the orexin neuron population in the male, firing rates were maximally diverse during ejaculation, similarly diverse though weaker during intromission, and inverse to this during anterior thrusting. In the female mouse, orexin neurons tended to decrease firing during intromission after a transient increase. We provide this brief dataset for re-use, to enable further studies of these rare behaviours with challenging surgical preparations.

## Introduction

The lateral hypothalamic area (LH) and its orexin/hypocretin (orexin) neurons are primarily known as vital controllers of arousal, feeding and metabolism [1,2]. However, early studies also showed that electrical stimulation of the LH rapidly and reversibly drives mating in the male rat [3]. In line with this, the orexin neurons integrate external and internal sensory information and output to most of the brain as well as the autonomic nervous system, and affect sex hormone secretion [1,4]. C-fos assays, pharmacological experiments and cell-specific lesion data suggest that orexin neurons in male rats are activated during mating, and orexin peptides and normal activity of orexin neurons facilitate mating initiation, performance and mating-induced place preference in male rats [5–8]. However, orexin neuron activity has not been recorded directly during mating, and therefore its roles in the appetitive and consummatory sequences of copulation are unknown. The activity of orexin neurons during mating is of particular interest as it has been suggested that a common side-effect of selective serotonin reuptake inhibitors (SSRIs), inhibition of mating, is mediated by increased serotonergic inhibition of orexin neurons [5,9–11].

Animal mating behaviours can be diverse, including in some species oral sexual encounters [12–15], which have not been documented in mice as far as we know. This lack of documentation would imply that the repertoire of rodent mating behaviours is limited to a canonical cycle of chasing, anogenital investigation, mounting and intromission [9,16,17]. One of the recordings here contains a type of oral sexual encounter, similar to fellatio, which we refer to as anterior thrusting (AT). Due to the low frequency of these behaviours (mating was observed in 6/22 recordings in two animals, and 0/13 recordings in three other animals) the findings are provided here as anecdotal evidence and data are provided for re-use. A subset of the presented recordings have been analyzed in a different way in our previous paper [18].

## Materials and Methods

### Animals

**Figure 1:**
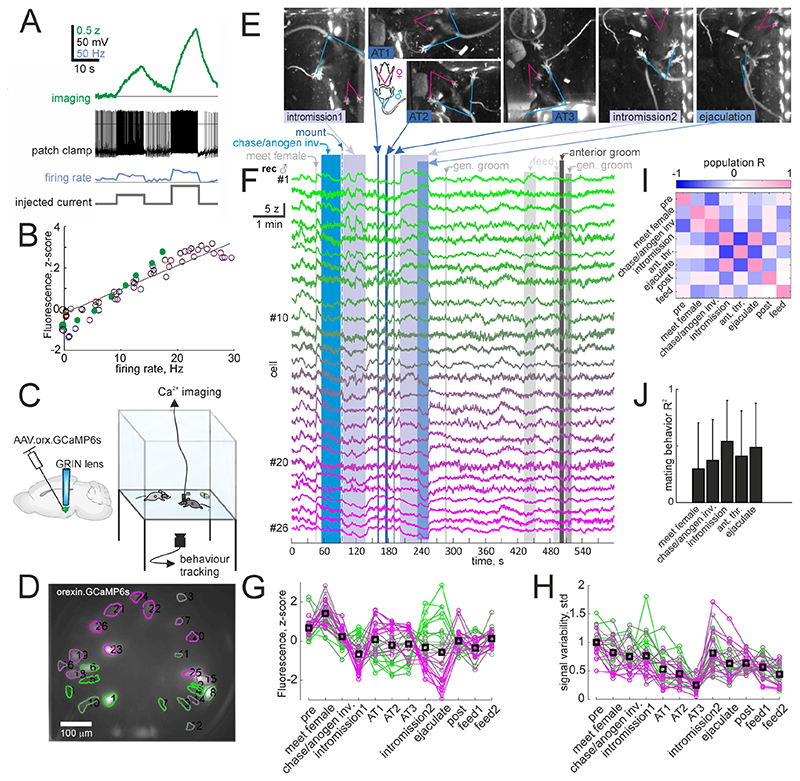
A, Example whole cell patch clamp recording while imaging an orexin-GCaMP6s neuron in a brain slice. B, Fluorescence to firing rate relationship indicating fluorescence recordings reliably report changes in orexin neuron firing rate. C, left, schematic of GCaMP6s delivery to orexin neurons and implantation of GRIN lens to obtain optical access to the neurons. Right, schematic of recording paradigm, with the subject mouse and an opposite sex conspecific in an enclosure with a glass bottom and a camera tracking behaviour from below. D, field of view of orexin neurons through the GRIN lens in the male mouse recorded in this figure, color coded same as F,G and H. E, example frames from behaviour video showing intromission, AT (anterior thrusting) and ejaculation epochs. The diagram shows annotation of male mouse rear paws and tail with a blue ‘V’ shape and female annotated from front paws to upper abdomen with a pink ‘V’ shape. F, Z-scored ΔF/F_0_ of the cells recorded during the social interaction during the 10th recording day for this subject. G, mean fluorescence of each cell during the indicated epochs. ‘Pre’ epoch is from the first time point to when the animals first meet, ‘post’ epoch is an equal time period after ejaculation. H, variability of the fluorescence signal expressed as the std during the same epochs as in G. I, correlation coefficients of the population signals across behaviour categories, showing inverse correlation between AT and intromission, and a positive correlation between intromission and ejaculation. J, mean coefficients of determination for each mating behaviour with the others. The color code for C, F, G and H are based on average firing rate during intromission as that has the overall highest coefficient of determination to the other behaviours shown in J.

**Figure 2:**
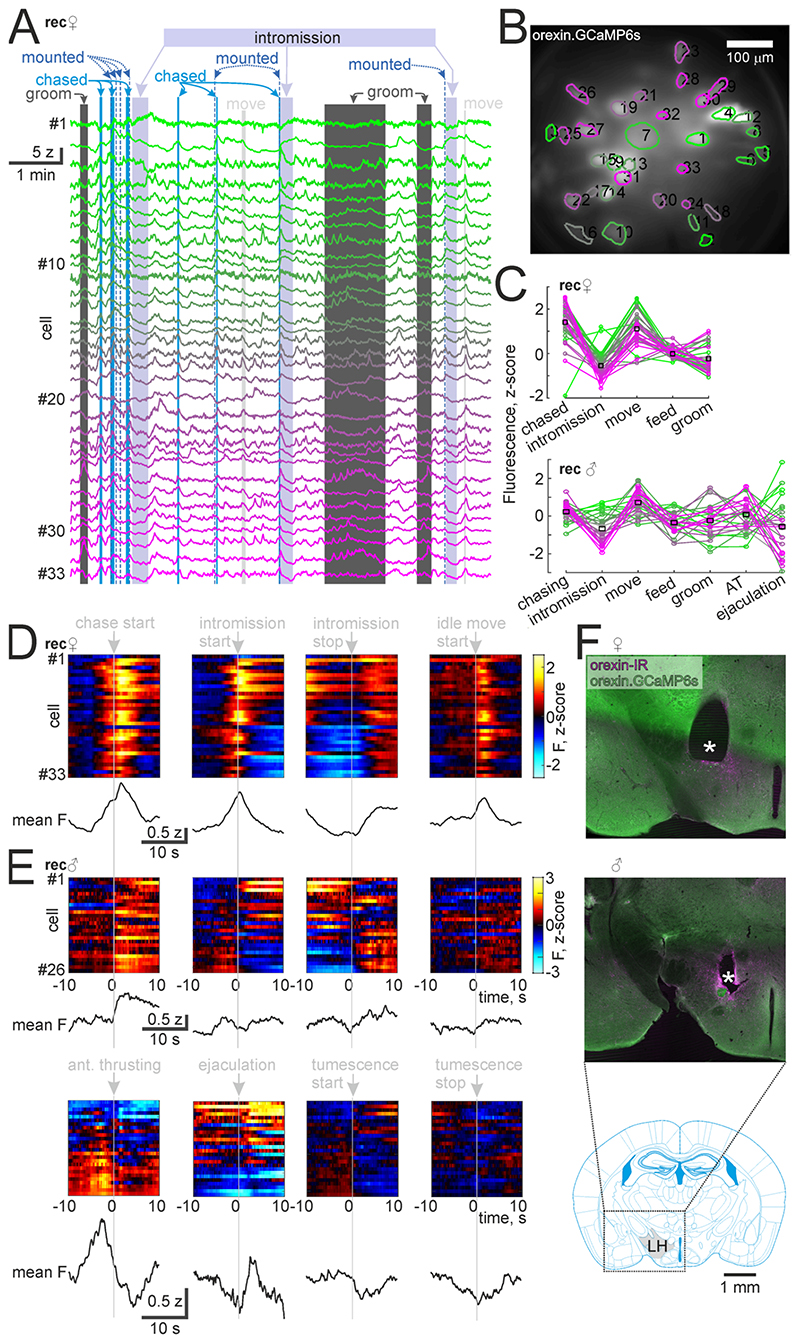
A, Z-scored ΔF/F0 of the cells recorded during the social interaction. B, field of view in the male mouse recorded in this A and C. C, mean fluorescence of each cell during the indicated epochs in the female recorded in A and B (above plot) and in the male recorded in Figure 1. D, event triggered averages of cell fluorescence across repetitions of the indicated behavioural events in the female. Mean across cells shown below the cell rasters. E, same as D but from the male recorded in Fig1, including also anterior thrusting, ejaculation, tumescence and detumescence of the penis. F, GRIN lens placements (* indicates the cylindrical lesion left by the GRIN lens) in the lateral hypothalamus in the female (above) and male (middle) along with an atlas schematic of the coronal plane (bottom).

Animal handling and experimentation was approved by the UK government (Home Office) and by Institutional Animal Welfare Ethical Review Panel. The recorded male was 86, the female was 79 and their conspecifics were 35 (female in Fig 1), 46 (different female used as conspecific for some data in Fig 2E) and 134 (sterile prm1 male in Fig 2A,B,D) days old at the beginning of the recordings. The recorded male had been in the recording arena with a female conspecific for 5 sessions (30-60min each, not supervised) prior to the first session where mating was recorded. It was therefore presumed sexually naïve at the first recording analysed here. Prior to the second session where mating was recorded (also the session with three AT epochs), the male was not entirely sexually naïve as it had been in 9 sessions with a female, out of which one had confirmed mating. The female conspecific in this recording was however sexually naïve. The recorded female had been in 12 sessions with a male before the recording shown here. In 3 of the prior sessions the female mated with the same conspecific as shown here. In between recordings, recorded mice were single housed in a controlled environment on a reversed 12h light-dark cycle with food and water ad libitum. Conspecifics were co-housed in groups of 2-5 same sex littermates.

### Surgery

Mice were stereotactically injected with AAV1-hORX.GCaMP6s (100-150nl, 2.5×10^12^ GC/ml, U Penn vector core). Orexin promoter virus expression specificity has been characterized previously [18]. After anesthesia with isoflurane, the scalp was infiltrated with lidocaine and cut. Then a craniotomy was drilled at 0.9 mm lateral, 1.4 mm posterior from Bregma. A pulled borosilicate glass injection tip was used to inject virus at a depth of 5.4 mm at a rate of 50 nl/min. The scalp was sutured or glued closed after removal of the injection tip. Animals received 5 mg/kg carprofen injections for two days as post-operative pain medication.

Two weeks after the virus injection, a custom-made aluminium head plate was attached to the skull with dental cement (Metabond) and skull screws. A 0.8 mm diameter hole was drilled at the same position as in virus injection surgery and a 0.39 NA, 7.3 mm long, 0.6 mm diameter cylindrical graded refractive index (GRIN) lens (Grintech) was lowered slowly (150 μm/min) to a depth of 5.1 mm with a micromanipulator. The GRIN lens was then cemented onto the skull. Thereafter the implant was coated with black dental cement and painted with several layers of black nail polish to protect from light contamination.

Animals received one dose of 0.6mg/kg dexamethasone as antiinflammatory medication along with 5mg/kg carprofen injections for post-operative pain management for two days. After a minimum of two weeks of recovery, mice were trained for freely moving miniature endoscope recordings under an incremental regimen of wearing the head-mounted miniature microscope (~2g weight) in the test arena (Figure 1C).

### Freely moving Ca^2+^ imaging

Ca^2+^ imaging was performed with a miniaturized head-mounted microscope (Inscopix) which was attached to the animal’s head, without anaesthesia shortly before recording. The animal was then placed into the arena with a plexiglass floor. The animal had already spent time in the same arena leaving scent markings. Recording was begun after approximately 10 minutes of initial exploration of the familiar arena. Each recording consisted of observation of the subject and conspecific with food and water available ad libitum. Sexual receptivity was not induced with hormones, females were not ovariectomized and estrous was not confirmed with vaginal cytology. The arena was lit with red LED lighting and was covered from view in a dark, quiet room with a silent fume extractor pipe above the arena to eliminate background odors. Ca^2+^ imaging frame rate was 10 frames/s. Behaviour was tracked with a CCD camera (Lumenera Infinity) from below the arena at 65 frames/s. An LED facing the behaviour camera and blinking in response to each Ca^2+^ imaging frame was used to synchronize Ca^2+^ imaging and behaviour videos. Behaviour analysis was performed in ImageJ by manually annotating behaviours. The full datasets are available for free re-use under a public domain dedication [19].

### Ca^2+^ imaging analysis

Ca^2+^ imaging data were preprocessed for dropped frames and pixels, downsampled by a factor of three spatially, then motion corrected in Mosaic (Inscopix) and saved as 16bit TIFF stacks. Further processing was performed in Matlab with custom routines. Regions of interest (ROIs) were drawn manually around cells and average fluorescence was extracted across all pixels within each ROI as well as a neuropil ‘halo’ around each ROI consisting of the third to the sixth nearest pixels outside the outline that did not contain other ROIs. These signals were lowpass filtered with a moving average of three frames. Neural signals were calculated by subtracting the neuropil ‘halo’ signal from each ROI specific signal. This signal was then corrected for photobleaching by computing ΔF/F_0_ using the mean over a 120 s moving window as F_0_ and z-scored.

### Patch-clamp recordings

Correspondence between firing rate and GCaMP6 fluorescence was recorded in whole cell mode as described in [18]. Briefly, acute slices were cut from animals injected with AAV1-hORX.GCaMP6s as above. A whole-cell recording was established and the same miniature microscope and GRIN lens as in the in vivo recordings, was placed close to the recorded cell. Then 10s current steps of varying size were injected intracellularly to elicit varying firing frequencies. The 0.3 mm brain slices were bathed in continuously bubbled (95% O_2_ and 5% CO_2_) ACSF, containing (in mM): 126 NaCl, 3KCl, 2MgSO_4_, 2CaCl_2_,1.1 NaH_2_PO_4_, 26 NaHCO_3_, 0.1 pyruvic acid, 0.5 L-glutamine, 0.4 ascorbic acid and 25 D-glucose. Intracellular solution contained (in mM): 130 K-gluconate, 5 NaCl, 2 MgSO_2_,10 HEPES, 0.1 EGTA, 4 Mg-ATP, 0.4 Na-GTP, 2 pyruvic acid, 0.1 Alexa-594, 0.1% biocytin, and 10 mM KOH (to set pH to 7.3).

### Histology

Following standard perfusions as described in [18], 0.1 mm sections were cut on a vibratome. Sections were blocked in PBS with 0.3% Triton X-100 and 1% bovine serum albumin (blocking solution) for 1 h, incubated with goat anti-orexin (1:1000; Cat# 8072, RRID: AB_653601, Santa Cruz) over-night, washed, incubated with Alexa 647 conjugated donkey anti-goat (1:1000; Cat# A-21447, RRID: AB_2535864, Invitrogen/Thermo Fisher Scientific) for 3.5 h, washed and mounted. Antibodies were applied in blocking solution. Confocal micrographs were acquired on a Nikon A1 and merged in imageJ.

### Statistics

All data are shown as mean ± std unless stated otherwise.

## Results

### Orexin neuron activity during intromission and AT in the male mouse, and during intromission in the female

We recorded orexin neuron firing rates using the GCaMP6s Ca^2+^ sensor [20] which was delivered in a viral vector under the orexin promoter yielding GCaMP6s expression with 97.4 ± 1.0 % specificity and 65.8 ± 3.7 % penetrance in orexin neurons [18]. Simultaneous electrical and optical recordings in brain slices showed that GCaMP6s fluorescence faithfully reported spiking activity (Figure 1A,B). Using implanted graded refractive index (GRIN, Figure 1C) lenses and a miniature fluorescence microscope mounted on the skull, we recorded activity of orexin neuron populations in freely behaving mice as they navigated a familiar 24x24cm arena that contained food pellets, water and an opposite sex conspecific (Figure 1C-F). Some of these datasets were analyzed in less detail in Figure 1 of our previous article [18], and here we report in more detail on the mating behaviours observed in a limited subset of those, and previously undocumented recordings.

The firing rate changes in male orexin neurons were large during mating behaviours, reaching, in different cells, peak and through values during ejaculation corresponding to the extremes of their 0 to 30 Hz firing range (Figure 1B,F,G). The population firing pattern of orexin neurons during AT was inverted compared to that during intromission (Figure 1G), and firing was less variable during AT (Figure 1H) than other mating behaviours. Correlation coefficients of the population firing rate vector across behaviours revealed that intromission and ejaculation were highly correlated and both were inversely correlated with AT (Figure 1I). Intromission had the strongest average of coefficients of determination (R^2^) with other mating behaviours (Figure 1J), and was therefore used to sort neurons in plots and color code.

AT was observed in the last one out of ten recordings in one male over an 86 day period. At this point the male was not sexually naïve, though the female was (see Methods). This behaviour consisted of the male approaching the female’s head from the front or side, rearing, using front paws to push down the female’s head followed by erection and multiple rapid thrusts of the penis into the female’s mouth (Movie 1, Figure 1E). AT occurred in three bouts each lasting 2.4,4.6 and 1.1 s and containing an estimated 21, 13 and 15 thrusts toward the mouth and 5, 4 and 9 estimated insertions of the penis into the female’s mouth. The bouts ended with the female rearing away or turning to the side, penile detumescence and either the male or female moving away while the other remained in place, apart from the third bout which ended in the male chasing the female and intromission. The multiple thrusts into the mouth and the coordinated end without signs of aggression, stress or escape suggest that the behaviour was not aversive. The bouts occurred during a 64.5 s interval between two bouts of intromission. The delay from stop of first intromission to start of first AT was 22.5 s. The intervals between AT bouts were 11.9 and 11.4 s and intromission started 10.9 s after the third AT bout. Intromission before AT lasted 41.6 s, whereas after the AT intromission lead to ejaculation in 31.5 s, which was evident as spasming followed by falling to the side and prolonged immobility (Figure 1E). In another recording 29 days prior, intromission also occurred but bouts were shorter (29.7 ± 26.8 s, n=4). In this recording there was one apparent attempted AT interrupted by the female rearing away. Total cumulative time of intromission before ejaculation was 73.1 s (across 2 bouts) with AT and 101.9 s (across 4 bouts) without AT. These data would suggest that mouse AT shortens mating duration by hastening ejaculation.

We also captured mating in a female mouse during orexin neuron imaging (Figure 2A-D). These recordings did not contain AT bouts. As a contrast to the male firing patterns, female orexin neurons were predominantly inhibited during intromission after an initial peak in their activity (Figure 2C-E). We additionally aligned the male neural activity to erections. On average, orexin neuron activity started to decrease upon onset of tumescence of the penis, and onset of detumescence coincided with the through of orexin neuron activity (Figure 2E). These data suggest that overall, copulation coincides with a decrease of orexin neuron firing in both sexes. However, both sexes had heterogeneity within the neural population, and in particular the male had several orexin neurons that increased activity during tumescence, intromission and ejaculation (Figure 2E). GRIN lens placement was confirmed to be in the lateral hypothalamus (Figure 2F).

## Discussion

This pilot study is an anecdotal report as it consists of two recordings in a male and a female mouse. The data however indicate that orexin neuron activity is strongly and heterogeneously modulated during mating behaviours, and suggest that mouse mating behaviours can exhibit more diversity than documented previously. Though AT seemed non-aversive to the female, it is possible that the size of the arena limited the options to retreat from this behaviour, as female mice pace mating behaviours when given the opportunity to retreat [21]. We should note that the head mounted miniature microscope is ~3 cm high and thus may affect mating behaviours, such as AT when the female is recorded. Another potential caveat is that the GRIN lens implantation entails lesioning brain tissue in somatosensory neocortex, dorsal hippocampus and thalamus in one hemisphere, as these structures stand in the path of the lens.

These recordings suggest that sub-second firing rate changes in orexin neurons have a role in mating behaviours. What could this role be? We recently reported that orexin neurons are involved in locomotion and sensorimotor processing [18]. The increased orexin neuron firing during chasing (Figure 2D,E) is likely a part of that. Orexin neurons may have a role in supporting the motor outflow underlying intromission as the female who typically remains stationary has overall more inhibited orexin neurons than the male, who is thrusting (Figure 2C-E). During penile tumescence (which coincided with thrusting) the male orexin neurons tended to decrease activity, suggesting that overall orexin neuron activity specifically increases locomotion [18] rather than other action patterns. Additionally orexin neurons may have a role in orchestrating autonomic nervous system outflow and CNS reward processing during ejaculation, as, during ejaculation, they express even more strongly their intromission firing rates (Figure 1F,G). This is in line with previous work showing decreased conditioned place preference for mating behaviour in male mice upon orexin neuron ablation [8]. AT lasted 2.7 ± 1.8 s while intromission lasted 35.6 ± 22.9 s (p=0.048 with Student’s t-test), suggesting an early switch to other behaviour patterns from AT. The inversion of the male intromission firing pattern during AT (Figure 1G,I,2E) could be part of a mechanism for switching of action plans. As orexin neurons promote wakefulness, their activity during mating may critically support arousal while the CNS is engaged in coordinating autonomic nervous system outflow. This is consistent with occasional reports of loss of consciousness during sex - orgasmolepsy - in narcoleptic patients, who typically lack orexin signaling [22].
